# Primary Small Cell Neuroendocrine Carcinoma of Paranasal Sinuses

**DOI:** 10.1155/2014/874719

**Published:** 2014-06-25

**Authors:** Maliha Khan, Sobia Nizami, Aibek E. Mirrakhimov, Benjamin Maughan, Justin A. Bishop, William H. Sharfman

**Affiliations:** ^1^Presence Saint Joseph Hospital, Department of Internal Medicine, 2900 N. Lake Shore Drive, Chicago, IL 60657, USA; ^2^Department of Medicine, Aga Khan University, Karachi, Sindh 74800, Pakistan; ^3^Sidney Kimmel Comprehensive Cancer Center, John Hopkins University, Baltimore, MD 21287, USA

## Abstract

Small cell neuroendocrine carcinoma of the paranasal sinuses is an extremely rare and aggressive neoplasm. Despite aggressive management, the tumor carries a poor prognosis, with a high risk of local recurrence or distant metastases. The management strategy is based on that for pulmonary small cell cancer and includes platinum-based chemotherapy combined with radiotherapy. We are reporting a case of an 89-year-old female patient diagnosed with small cell carcinoma of right-sided ethmoid and sphenoid sinuses. The tumor was found to have invaded the right orbit and anterior cranial fossa. Metastases to cervical lymph nodes and bone were also found. Due to the extended stage and poor prognosis of the patient, the management plan is palliative chemoradiotherapy.

## 1. Introduction

Small cell carcinoma (SCC) is a poorly differentiated neuroendocrine tumor that most commonly occurs in the lung [1]. Small cell neuroendocrine carcinoma (SNEC) of the paranasal sinuses is an extremely rare and aggressive tumor that demonstrates rapid expansion and early hematogenous spread [2]. Although the tumor is responsive to initial local therapy [1], it is associated with frequent local recurrence and new distant metastases, leading to poor prognosis [2]. Here we report the case of a patient diagnosed with small cell neuroendocrine carcinoma of the ethmoid/sphenoid sinuses with local intracranial extension, who was referred to our center for management.

## 2. Case History

**Figure 1:**
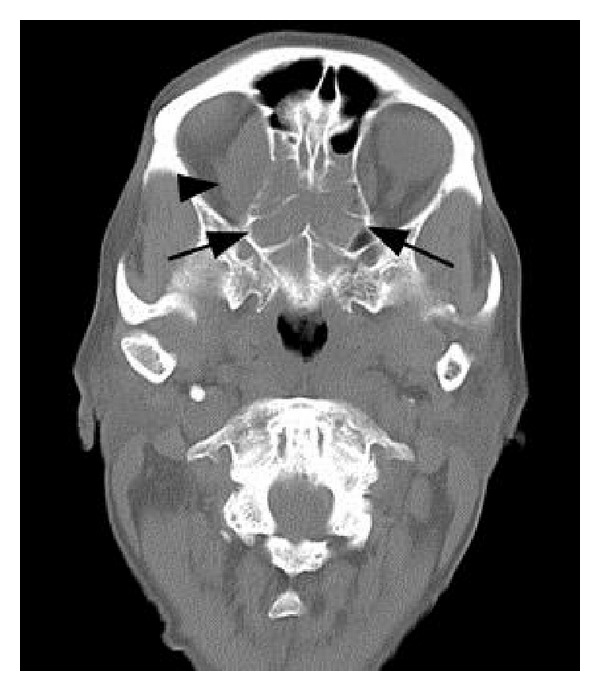
Axial noncontrast brain CT (bone algorithm image) showing a large sinonasal mass involving the ethmoid/sphenoid minuses (arrows) with right orbital extension (arrowhead).

**Figure 2:**
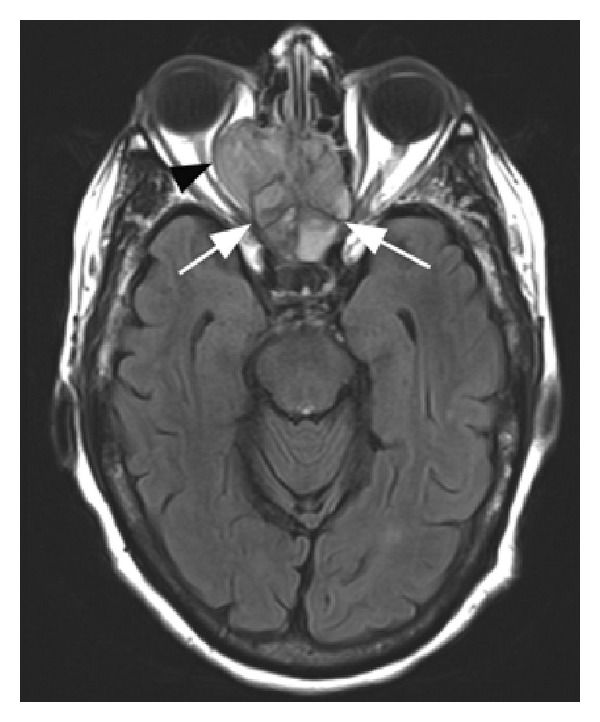
Axial FLAIR T2-weighted brain MRI showing a sinonasal mass involving the ethmoid/sphenoid sinuses (white arrows) with extension into the right orbit (black arrowhead).

**Figure 3:**
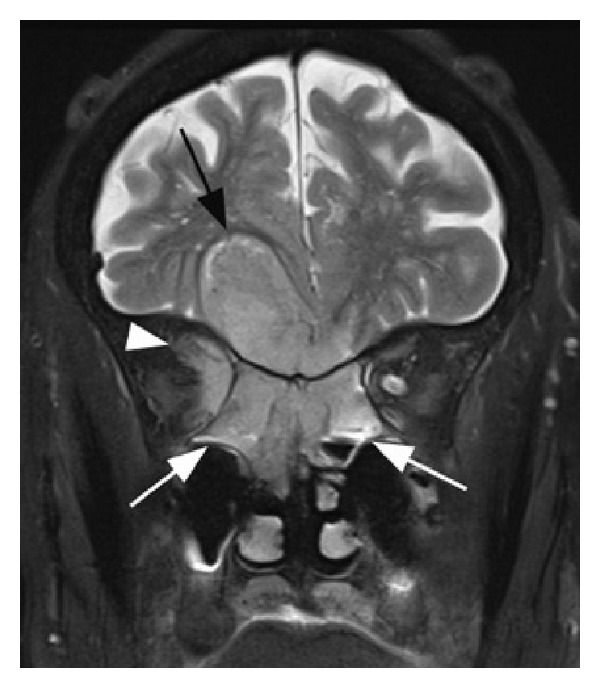
Coronal T2-weighted brain MRI showing a sinonasal mass involving the ethmoid/sphenoid sinuses (white arrows) with extension into the right orbit (white arrow head) and anterior cranial fossa (black arrow).

**Figure 4:**
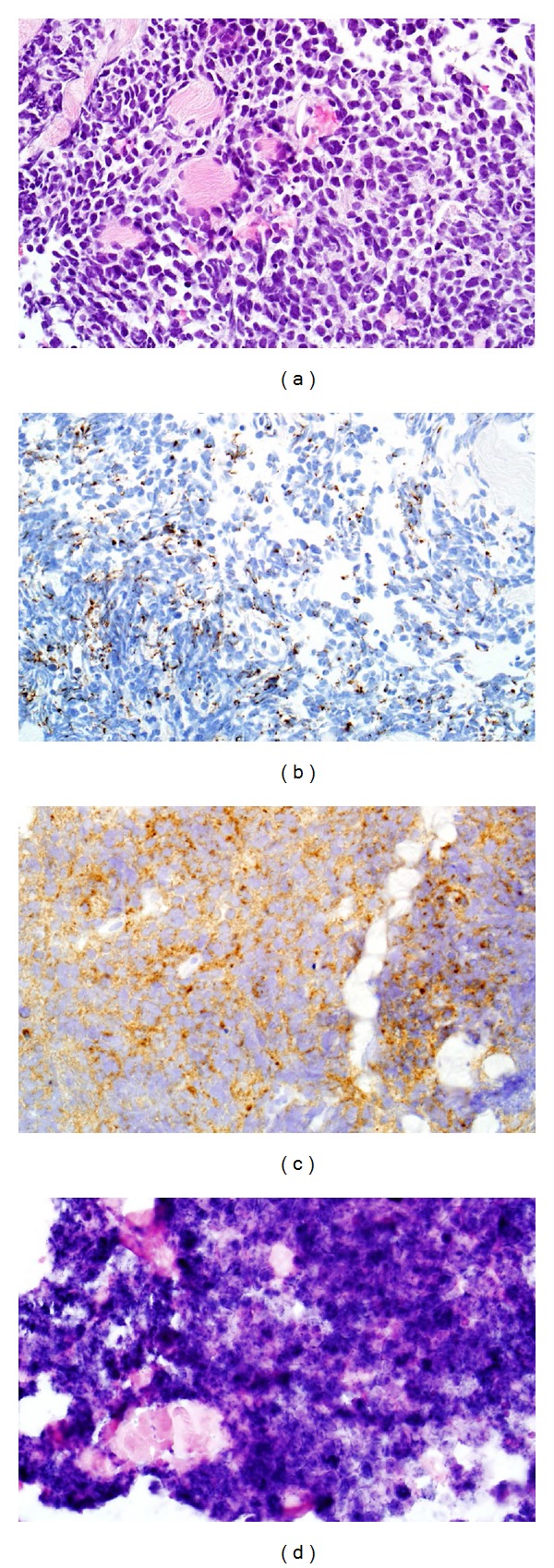
Hematoxylin and eosin staining showing nasal mass with small cell carcinoma (a), immunohistochemical staining showing tumor positivity for AE1/AE3 in a dot-like pattern (b), synaptophysin (c), and high-risk HPV by in situ hybridization (d). (a) The tumor consisted of a uniform population of small cells with minimal cytoplasm and hyperchromatic, angulated nuclei that demonstrated molding. The tumor exhibited high grade features including a high mitotic rate and necrosis (hematoxylin and eosin, ×400). (b) The neoplasm was positive for cytokeratin on a perinuclear, dot-like pattern (AE1/AE3 immunohistochemistry, ×400). (c) The tumor exhibited neuroendocrine differentiation in the form of diffuse staining for synaptophysin (synaptophysin immunohistochemistry, ×400). (d) HPV studies showed that the tumor harbored high-risk HPV DNA (high-risk HPV in situ hybridization, ×400).

An 89-year-old female with a history of hypertension, hypercholesterolemia, glaucoma, and osteoarthritis presented with a history of headaches and facial pain over the right side of her face for one month. Other symptoms included decreased vision in the right eye, intermittent diplopia, difficulty in closing the right eye, and gait unsteadiness. She described having overall fatigue and lower back pain, worse on walking, for the past 3 months. There was no past history of cigarette smoking. Physical examination was positive for complete right eyelid closure, no overlying skin changes and no cervical lymphadenopathy. Brain CT and MRI scans revealed a large sinonasal mass involving the ethmoid/sphenoid sinuses and extending into the right orbit and anterior cranial fossa (Figures 1, 2, and 3). Fine-need aspiration of the nasal mass confirmed the tumor as small cell carcinoma (Figure 4(a)). A PET scan was done to look for distant disease as well as a possible lung primary, which showed multiple bone metastases but no lung mass. Immunohistochemical studies demonstrated tumor positivity for AE1/AE3 in a dot-like pattern (Figure 4(b)), synaptophysin (Figure 4(c)), and chromogranin and were negative for CD45, S100, and myogenin. The tumor was also positive for high-risk HPV by in situ hybridization (Figure 4(d)). The patient was diagnosed with extensive stage small cell carcinoma of the ethmoid/sphenoid sinuses. Due to disseminated disease, she was scheduled for palliative chemotherapy with 6 cycles of carboplatin and etoposide every 21 days [3].

Given the extent of her disease proximal to the orbit, she was started on chemotherapy urgently as inpatient for the first cycle. So far, she has completed 3 cycles of carboplatin/etoposide. Her complications have been mild but include fatigue that peaked with her second cycle of chemotherapy. The fatigue has improved with initiation of packed red blood cell transfusions to treat her chemotherapy induced anemia. Her baseline hemoglobin level was 12 g/dL and decreased to 9 g/dL prior to transfusions. She also developed acute renal insufficiency with a creatinine of 1.6 mg/dL, increased from a baseline of 1.0 mg/dL previously. This developed after the third cycle of chemotherapy. The creatinine returned to baseline with intravenous fluids. Regarding the presenting symptoms of headaches and vision disturbances, these have decreased but not entirely resolved. The headaches are less frequent and now only mildly impaired. A repeated head MRI was done after the third cycle that demonstrates a very good partial response at the site of the primary lesion. The ethmoid lesion has entirely resolved and around 50 percent reduction in the size of the inferior frontal lobe mass. There is still a slight enhancement noted on the orbital nerve but otherwise the lesion in that region has regressed. A restaging PET scan for systemic response is pending at this time.

## 3. Discussion

Carcinoma of the nasal cavity and paranasal sinuses is an uncommon tumor, with an incidence of less than 1 per 100,000 persons per year [4]. Extrapulmonary small cell neuroendocrine carcinomas (EPSNEC) are rare, making up 0.1–0.4% of all cancers [1]. Only 11% of EPSNC occur in the head and neck [1]. Among EPSNEC, primary SNEC arising in the nasal sinuses is extremely rare. It was first reported as a differentiated histological type in the paranasal sinuses by Raychowdhuri in 1965 [5]. In the past 45 years, 76 cases of small cell neuroendocrine carcinoma of nasal and paranasal region have been described in the medical literature [6].

According to the World Health Organization criteria, small cell carcinomas are defined as malignant epithelial tumors consisting of small cells with scant cytoplasm, ill-defined borders, granular nuclear chromatin, absent nucleoli with extensive necrosis, and high mitotic count [7]. The tumor often stains positive for neuroendocrine markers such as synaptophysin, CD 56, and chromogranin A [8]. Small cell carcinomas of pulmonary and extrapulmonary origin all have similar morphologic, immunohistochemical, and ultrastructural features [9]. Due to the rarity of occurrence of EPSCC in the sinonasal tract, its initial workup must include searching for a primary tumor at a more likely site such as the lung [3].

EPSCC of the sinonasal tract has a slight male predominance. It can occur in any age group, and the mean age at presentation is 51–58 years [2, 8]. Despite the biological similarities between pulmonary and extrapulmonary SCC, the risk factors are not identical. Although a history of tobacco use correlates strongly with pulmonary small cell carcinoma, it has not been reported as a risk factor for EPSCC [2].

Human papilloma virus (HPV) infection is a possible risk factor for small cell carcinoma in the sinonasal tract [10]. High-risk positivity for HPV infection was found in 1 out of 6 cases of sinonasal small cell carcinoma in one retrospective study by Bishop et al. [10] and was seen in our patient as well. However, it is unclear whether HPV has any role in the pathogenesis of sinonasal small cell lung cancer.

Furthermore, paraneoplastic syndromes, seen in small cell lung cancer, are not common with EPSCC [11]. EPSCC can be classified as limited or extensive stage based on the extent of tumor spread. Local disease is defined as tumor confined to the primary site and regional lymph nodes, while tumors extending beyond locoregional boundaries are classified as extensive stage [8]. Despite aggressive management, the tumor carries a poor prognosis, with an overall 80% risk of local recurrence or distant metastases within 2 years after treatment [11]. The reported median survival for limited disease is 1.4 years, and for extensive disease it is 0.7 years [3]. The 5-year survival for limited disease is 25.4% and that for extensive disease is 0%. Occasionally, cases of limited stage sinus SCC have also been reported to be recurrence-free after 7–10 years of follow-up [11, 12].

Due to the rarity of the disease, clinical trials to identify the optimal management for EPSCC have not been done. Retrospective studies and case reports consistently recommend that EPSCC be managed similar to pulmonary SCC [3]. This includes combination chemotherapy with at least 4 cycles of cisplatin and etoposide, along with radiotherapy of at least 50 Gy in 2 Gy fractions [3]. Surgical intervention is usually reserved for treatment-resistant cases [13], or cases where complete tumor resection can be achieved with minimal morbidity [3]. Keeping in mind the poor prognosis of extensive disease in our patient, the management plan is palliative platinum-based chemotherapy.

## 4. Conclusions

In conclusion, the patient in this case was an 89-year-old female who presented with headaches and visual disturbances due to a sinonasal mass with intracranial extension. Biopsy revealed small cell carcinoma of the ethmoid and sphenoid sinuses of the right side, and further investigations demonstrated lymph node and multiple bone metastases. Due to the advanced stage and extensive spread of the tumor, the prognosis in this case is very poor, with an estimated survival of less than 2 years [3]. Therefore, the management for this patient is palliative chemotherapy with 6 cycles of carboplatin and etoposide.
